# Typical and extreme weather datasets for studying the resilience of buildings to climate change and heatwaves

**DOI:** 10.1038/s41597-024-03319-8

**Published:** 2024-05-23

**Authors:** Anaïs Machard, Agnese Salvati, Mamak P. Tootkaboni, Abhishek Gaur, Jiwei Zou, Liangzhu Leon Wang, Fuad Baba, Hua Ge, Facundo Bre, Emmanuel Bozonnet, Vincenzo Corrado, Xuan Luo, Ronnen Levinson, Sang Hoon Lee, Tianzhen Hong, Marcelo Salles Olinger, Rayner Maurício e Silva Machado, Emeli Lalesca Aparecida da Guarda, Rodolfo Kirch Veiga, Roberto Lamberts, Afshin Afshari, Delphine Ramon, Hoang Ngoc Dung Ngo, Abantika Sengupta, Hilde Breesch, Nicolas Heijmans, Jade Deltour, Xavier Kuborn, Sana Sayadi, Bin Qian, Chen Zhang, Ramin Rahif, Shady Attia, Philipp Stern, Peter Holzer

**Affiliations:** 1 Scientific and Technical Building Research Centre (CSTB), Energy and Environment Department, Grenoble, France; 2 Laboratory of Engineering Sciences for the Environment (LaSIE), La Rochelle University, La Rochelle, France; 3 Architecture, Energy & Environment (AiE), Polytechnic University of Catalonia, Barcelona, Spain; 4 Brunel University London, London, UK; 5 Department of Energy, Politecnico di Torino, Torino, Italy; 6 National Research Council Canada, Ottawa, Canada; 7 Centre for Zero Energy Building Studies, Department of Building, Civil and Environmental Engineering, Concordia University, Montreal, Canada; 8 Faculty of Engineering, British University in Dubai, Dubai, UAE; 9 Institute of Construction and Building Materials, Technical University of Darmstadt, Darmstadt, 64287 Germany; 10 Centro de Investigación de Métodos Computacionales (CIMEC), UNL/CONICET, Santa Fe, 3000 Argentina; 11 Lawrence Berkeley National Laboratory (LBNL), Berkeley, California USA; 12 Laboratory for Energy Efficiency in Buildings, Federal University of Santa Catarina, Florianopolis, Brazil; 13 Fraunhofer Institute for Building Physics IBP, Valley, Germany; 14 Department of Architecture, Faculty of Engineering Science, KU Leuven, Belgium; 15 Building Physics and Sustainable Design, Department of Civil Engineering, KU Leuven, Belgium; 16 Department of Environmental Architecture, Faculty of Architecture and Planning, Hanoi University of Civil Engineering, Ha Noi, Viet Nam; 17 Buildwise, Brussels, Belgium; 18 University of Gävle, 801 76 Gävle, Sweden; 19 Department of the Built Environment, Aalborg University, Aalborg, Denmark; 20 China Southwest Architecture Design and Research Institute Corp. Ltd., Chengdu, China; 21 Sustainable Building Design Lab, Department UEE, Faculty of Applied Sciences, Université de Liège, Liège, Belgium; 22 Institute of Building Research and Innovation (IBR&I), Wien, Austria

**Keywords:** Mechanical engineering, Civil engineering, Energy infrastructure

## Abstract

We present unprecedented datasets of current and future projected weather files for building simulations in 15 major cities distributed across 10 climate zones worldwide. The datasets include ambient air temperature, relative humidity, atmospheric pressure, direct and diffuse solar irradiance, and wind speed at hourly resolution, which are essential climate elements needed to undertake building simulations. The datasets contain typical and extreme weather years in the EnergyPlus weather file (EPW) format and multiyear projections in comma-separated value (CSV) format for three periods: historical (2001–2020), future mid-term (2041–2060), and future long-term (2081–2100). The datasets were generated from projections of one regional climate model, which were bias-corrected using multiyear observational data for each city. The methodology used makes the datasets among the first to incorporate complex changes in the future climate for the frequency, duration, and magnitude of extreme temperatures. These datasets, created within the IEA EBC Annex 80 “Resilient Cooling for Buildings”, are ready to be used for different types of building adaptation and resilience studies to climate change and heatwaves.

## Background & Summary

Climate change is among the most significant challenges the global community faces in the 21^st^ century, with direct consequences for the building sector. An increase in the magnitude, frequency, and intensity of natural hazards presents a threat to the structural integrity of the buildings. In contrast, changes in climate characteristics, such as rising temperatures and more frequent extreme heat events, present an unprecedented challenge to building designers to design buildings that can perform efficiently over their durations of use. The performance evaluation of renovated or new buildings should consider not only the current average and extreme climates but also expected future climates and extreme events. To achieve this aim, reliable weather files capturing present, future typical, and extreme weather conditions are necessary to carry out building and resilience strategies studies. To reduce the computational costs associated with running building simulation models over long periods of time, simulations are generally performed over subsets of long-term climate data, typically over one year, referred to as reference meteorological years. Depending on the application, either a typical meteorological year (TMY) or an extreme meteorological year (XMY) is chosen. Many researchers and building practitioners are currently using only future TMYs to assess the impact of climate change on building energy performance because future TMYs are easily accessible and usually built from simplified statistical methods to account for climate change (e.g., the morphing method from Belcher *et al*.^1^). Although morphing offers a quick way to generate weather files, it does not account for complex future changes in climate variables, such as changes in the frequency and duration of extreme heat events. Therefore, the generation of future weather files containing extremes has been an ongoing challenge for the building community in the last decade. A few authors have started to use climate model outputs directly to prepare the building simulation weather files to assemble not only future TMYs but also future extreme weather files such as heatwave events (HWE) or extreme meteorological years (XMYs). For example, Nik^2^ prepared typical and extreme weather files for Stockholm and Geneva. The typical and extreme years were selected solely based on the temperature parameter. These weather files were prepared from raw regional climate model (RCM) data from four different climate models without bias correction. Machard *et al*.^3^ prepared typical TMY and future HWE for France using data from four RCM and the Representative Concentration Pathway (RCP) 8.5 at 12.5-km spatial resolution. In Machard^4^, bias-adjustment of the RCM projections was added to the method. The typical years were assembled following ISO EN 15927-4^5^, giving equivalent weight to temperature, humidity, and solar irradiance and secondary weight to wind speed. The heatwaves were selected following the French national heatwave definition, based on daily daytime and nighttime temperatures above specific thresholds validated for France using a CORDEX dataset by Ouzeau^6^. Doutreloup *et al*.^7^ and Ramon *et al*.^8,9^ used a convection-permitting climate model at 2.8 km resolution driven by the EC-Earth RCM and coupled with the land-surface scheme TERRA_URB. Based on the bias-adjusted data^9,10^, they prepared TMYs for different locations in Belgium for an RCP 8.5 climate change scenario. They also prepared XMYs, selecting extreme months based on two parameters: temperature and solar irradiance. Gaur *et al*.^11,12^ used the Canadian RCM bias-corrected climate projections to prepare TMYs, typical and extreme moisture reference years, typical downscaled years, and extreme warm and extreme cold years for over 500 locations. Recently, Bass *et al*.^13^ published future TMYs for 18 cities in the United States based on six climate models and different socioeconomic scenarios, Shared Socioeconomic Pathways (SSP) 5 and RCP 8.5. The TMYs were assembled using data from six climate models to reduce individual model bias.

### Study scope

**Fig. 1:**
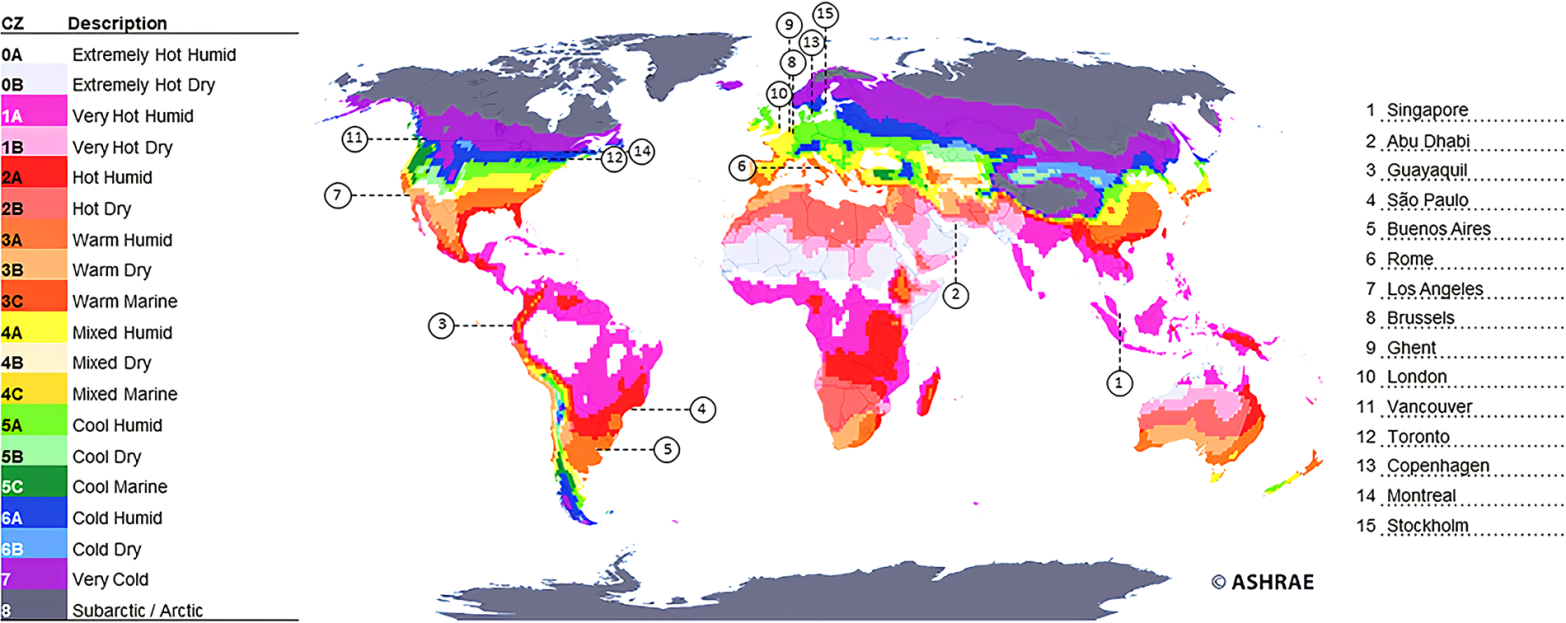
15 locations selected and ASHRAE 169–2013 climate classificatio*n*^14^.
- the future weather files are prepared directly from regional climate model simulation results and hence are able to account for complex future changes such as heatwaves in the climate variables projected for each city;
- the use of a multivariate bias-correction method is employed to correct the bias associated with the regional climate model simulations;
- the reference typical years and extreme heatwave event files are provided for building energy and overheating applications; and
- bias-corrected multi-year projections are also made available for additional research and other applications.

Future weather files based on bias-corrected RCM predictions are not easily available to the building scientific community; therefore, a large-scale international collaborative effort was made to curate and produce extreme weather data covering major global cities subject to extreme heat hazards by adopting a standardized procedure. This study prepares building simulation weather files ready to be used by building researchers and practitioners to carry out building energy simulations that are novel in the following respects:

- they have been prepared to employ a consistent methodology over 15 cities distributed across the globe in different continents and climate types for 10 climate zones worldwide, as defined by the American Society of Heating, Refrigerating and Air-Conditioning Engineers (ASHRAE) 169–2013^14^ (Fig. 1);

These datasets were developed for “Annex 80: Resilient Cooling of Buildings”, a research project of the International Energy Agency (IEA) - Energy in Buildings and Communities Programme (EBC)^15^, to evaluate the resilience of different passive and active cooling strategies.

They are used within the framework defined in Attia *et al*.^16^ and applied in Rahif *et al*.^17^. These weather files are shared to conduct climate change adaptation studies such as overheating risk assessments or a rise in demand for air conditioning under future typical and extreme weather conditions. The multi-year dataset is provided in comma-separated values (CSV) format so that it can easily be used for adaptation studies in other fields of investigation.

### Selected cities

The weather datasets have been generated for 15 cities representative of the ten climate zones of ASHRAE classification^14^. Cities were selected to include at least one city per zone in climate zones 0 to 6 because climate change is expected to markedly increase cooling demand in these zones^18^. Preference was given to cities with high populations and high population growth. Most are in Europe, North America, and Asia due to the limitations of gathering observational data for other locations. However, these are also the continents where the most heatwave events have been recorded in the last decade^19^. The cities of interest and population data are presented in Table 1.

**Table 1:** Cities analyzed and population data^96^.

ASHRAE Climate Zone (CZ)	City	Population 2022 (M)	Change % (since 2021)	Country	Continent
0A	Singapore	6.0	0.80%	Singapore	Asia
0B	Abu Dhabi	1.5	1.86%	UAE	Asia
1A	Guayaquil	3.1	1.62%	Ecuador	South America
2A	Sao Paulo	22.4	0.86%	Brazil	South America
3A	Buenos Aires	15.4	0.74%	Argentina	South America
3A	Rome	4.3	0.47%	Italy	Europe
3B	Los Angeles	4.0	0.05%	USA	North America
4A	Brussels	2.1	0.67%	Belgium	Europe
4A	Ghent	0.3	0.48%	Belgium	Europe
4A	London	9.5	1.22%	UK	Europe
4C	Vancouver	2.6	0.97%	Canada	North America
5A	Toronto	6.3	0.93%	Canada	North America
5A	Copenhagen	1.4	0.85%	Denmark	Europe
6A	Montreal	4.3	0.68%	Canada	North America
6A	Stockholm	1.7	1.36%	Sweden	Europe

## Methods

**Fig. 2:**
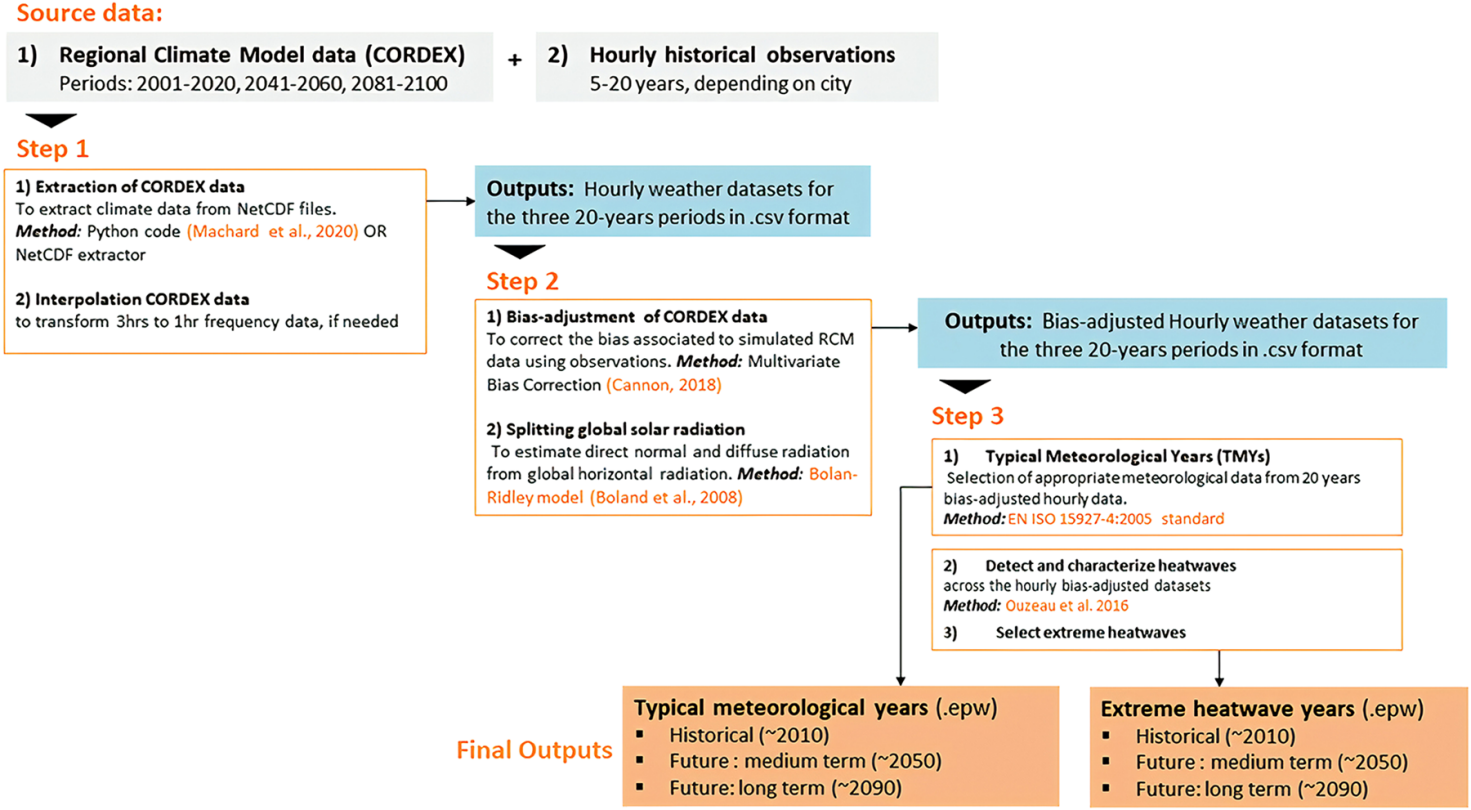
Methodology used for the weather datasets generation.

The flow chart in Fig. 2 illustrates the steps adopted to generate the weather files. In step 1, raw climate data were extracted for the different weather variables that dominantly affect the thermal performance of buildings for historical and two future periods (20 years for each period). In step 2, these raw climate data were bias-corrected using observations of the different weather variables for the specific locations. In step 3, the weather files were assembled from the multiyear bias-adjusted datasets to generate (a) TMYs based on the EN ISO 15927-4 standard^5^ and (b) heatwave years (HWYs), based on the method to detect the heatwaves on a CORDEX dataset proposed by Ouzeau *et al*.^6^, already tested for building performance simulations in^20^. Our methods are detailed in the following sections.

### Boundary conditions

The historical and future projected climate simulations needed to prepare the weather files were taken from the Coordinated Regional Downscaling Experiment (CORDEX)^21,22^ results contributed by the scientific community towards the Coupled Model Intercomparison Project 5^th^ Phase (CMIP5^23^). The CORDEX climate datasets for CMIP6^24^ were not available at the time our datasets were being prepared, so they were not considered. The future projections made under the Representative Concentration Pathway (RCP) 8.5 were considered^25^. RCP 8.5 is the highest baseline scenario in which emissions rise throughout the twenty-first century. In this scenario, the emissions and concentrations of greenhouse gases rise significantly over time, causing a radiative forcing of 8.5 W/m² by the end of the century^26^. This scenario is the most conservative greenhouse gas emission scenario of the Coupled Model Intercomparison Project 5^th^ Phase (CMIP5) which is also in line with the current emission trajectories of greenhouse gases around the globe^27^ and therefore RCP 8.5 was chosen to evaluate the worst case possible in a resilience and adaptation context.

**Fig. 3:**
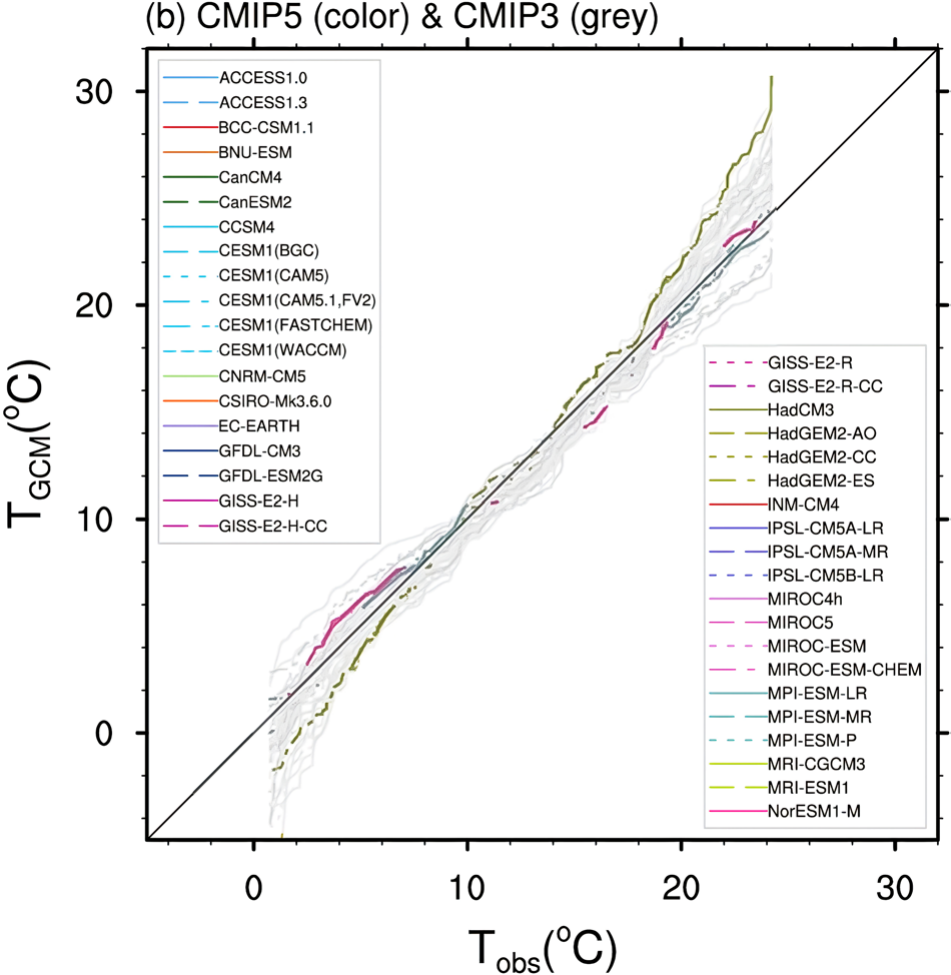
Selection of the climate model to generate future weather datasets – Position of the temperature projection from HadGEM2-ES, MPI-ESM-LR, and NorESM1-M in comparison with other model climate projections. Modified from: Flato, Gregory, *et al*. ‘Evaluation of climate models.’ Climate change 2013^69^.

To select an appropriate climate simulation from the CORDEX database, i.e., containing data for many different General Circulation Model (GCM) and Regional Climate Model (RCM) combinations, we referred to the findings of McSweeney *et al*.^28^. These authors analyzed all GCMs participating in the CORDEX database, and three reliable GCMs with low, medium, and high global equilibrium climate sensitivity (ECS) were identified as NCC-NORESM (Norwegian Earth System Model, developed by the Norwegian Climate Center), MPI-ESM-LR (Max Planck Institute Earth System Model for the High-Resolution Model), and HadGEM-ES (Hadley Centre Global Environment Model with an Earth-System configuration), respectively. These three GCMs have also been used to conduct coordinated downscaling experiments in CORDEX CORE simulations^29^. In addition to this, we conducted a review of available CORDEX simulations at the needed temporal frequency (sub-daily) across different CORDEX domains encompassing the different cities we are analyzing. The dry-bulb temperature projections of these three climate models were compared with reference to the evaluation of the climate models report (contribution of Working Group I to the IPCC AR5). Finally, the MPI-ESM-LR (GCM) and REMO (RCM) combination was selected for this work as it was associated with medium global ECS, was found to be the closest to the median temperature of all climate model projections (Fig. 3) and contained simulations in the required temporal frequency (at least 3-hourly or more frequent) for all domains. This selected simulation is henceforth referred to as “MPI-REMO”.

### Downscaled climate simulations

The selected GCM, MPI-ESM-LR^30^, is dynamically downscaled by means of an RCM, REMO^31,32^. REMO is a three-dimensional atmosphere model developed at the Max Planck Institute for Meteorology in Hamburg, Germany, and currently maintained at the Climate Service Center Germany (GERICS) in Hamburg. The model is based on the Europa Model, the former NWP model of the German Weather Service. The prognostic variables in REMO are horizontal wind components, surface pressure, air temperature, specific humidity, cloud liquid water, and ice. The physical packages originate from the global circulation model ECHAM4^33^, although many updates have been introduced^34–41^.

The MPI-REMO simulations, summarized in Table 2, were of 12.5 km spatial resolution for the European domain and 25 km resolution for other domains.

**Table 2:** Climate projections (model, scenario, spatial, and time frequency) used for each location.

Continent	Domain	Driving model	Downscaling method	Socio-economic scenario	Time frequency
Africa	AFR-22	MPI-ESM-LR	REMO 2015	RCP 8.5	3 HOURS
Asia	SEA-22				3 HOURS
Europe	EU-11				1 HOUR
South America	SAM-22				3 HOURS
North America	NAM-22				3 HOURS

RCM files were stored for each weather variable and for one year on the entire domain grid (a domain usually corresponds to an entire continent or parts of a continent) in NETCDF4 format. A Python code provided with this dataset was used to download the different NETCDF4 files, extract the nearest point to each city coordinates, and assemble the different weather variables and years in a single dataset. For each city, the weather variables downloaded are described in Table 3. They include dry-bulb temperature, specific or relative humidity, atmospheric pressure, surface downwelling shortwave irradiance, wind speed, and cloud cover (only for Europe). Additional variables, such as rainfall, wind direction, or longwave irradiance, are also important, but they were not available for all the cities; therefore, they were not used. Data were downloaded for the three time periods referenced in Table 4.

**Table 3:** Weather variables downloaded from the CORDEX platform.

EUR 11 Domain	AFR 22, NAM 22, SAM 22, and SEA 22 Domains
**tas** (near-surface air temperature)	**tas** (near-surface air temperature)
**hurs** (near-surface relative humidity)*	n/a
	**huss** (near-surface specific humidity)*
**ps** (surface air pressure)	**ps** (surface air pressure)
**rsds** (surface downwelling shortwave irradiance)	**rsds** (surface downwelling shortwave irradiance)
**clt** (total cloud fraction)	n/a
**sfcWind** (near-surface wind speed)	**sfcWind** (near-surface wind speed)
**clt** (cloud cover)	

*hurs is required in weather files for building performance simulations but was available only for the EU and SAM domains. For the other domains, the huss and tas variables are used to recalculate the hurs.

**Table 4:** 20-year periods downloaded for each variable from the CORDEX platform.

Period Name	Years
Historical – 2010s	2001–2020
Mid-term future – 2050s	2041–2060
Long-term future – 2090s	2081–2100

### Bias correction of climate model simulations

Climate model simulations are known to have bias associated with them because of the coarse spatial resolution at which the global or regional climate simulations are conducted^42^. The biases in the climate simulations, if left uncorrected, have been known to lead to incorrect descriptions of climate-driven hazards, such as floods^43^ and wildfires^44^. Many bias-correction methods have been discussed in literature^42^. The complexity of the methods can range from methods correcting simply the mean bias^45^ to methods able to perform univariate and multivariate distribution-based corrections^46^. The multivariate bias-correction methods have been found most efficient in correcting bias in the marginal distribution of the climate variables, as well as the inter-relationships between the variables, and have been recommended for accurately describing hazards dependent on multiple climate variables^46^. Therefore, the bias correction of raw climate variables was performed using quantile delta mapping (QDM)^47^ and Multivariate Bias Correction with N-dimensional probability density function transform (MBCn)^46^ methods. The QDM is a univariate bias-correction method that preserves climate model projected future changes in the quantiles of climate variables while at the same time correcting systematic biases in the quantiles. Climate model data are de-trended and then mapped onto the observations using quantile mapping. After that, future projected bias-corrected datasets are obtained by multiplying/adding to them the climate model projected future relative/additive changes in quantiles. The MBCn method extends the application of the QDM method in a multivariate context. First, individual climate variables are corrected following the QDM method. Thereafter, the dependence structure of climate variables is corrected using an iterative reshuffling process where, in each iteration, climate data are rotated by multiplying them with random orthogonal matrices, QDM is corrected and then re-correlated using inverse random matrices.

While all climate variables were bias corrected using the MBCn method, the QDM method was used to correct global solar irradiance because our analysis shows that the reshuffling of marginally corrected global solar irradiance values, as performed in the MBCn method, breaks the diurnal structure of global solar irradiance. This can subsequently lead to unrealistic values for not only global solar irradiance but also direct and diffused solar irradiance components derived from it. The calibration of MBCn/QDM methods and subsequent prediction of bias-corrected values were performed individually for each month of the year to preserve month-to-month variability in bias-corrected climate data. The methods assume that the bias is the same in the future as in the present. All years with observational data available in different cities were considered for the calibration of bias-correction methods. The length of the observational period and the variables available for each city are reported in Table 5. The observational datasets included hourly values of air temperature (tas), relative humidity (hurs), global horizontal irradiation (rsds), wind speed (sfcWind), atmospheric pressure (ps), and cloud cover (clt). Just for Sao Paulo, for which hourly values could not be found for all weather variables, the hourly values of global horizontal irradiation and wind speed were derived from daily values. Hourly values for irradiation were calculated using the Zhang-Huang solar model^48^. The regression coefficients in the model were calibrated based on the daily values using a least-squares approximation. Hourly values for wind speed were obtained by adjusting the monthly cumulative frequency distributions of historical RCM data to the observational data. Hence, each day of the RCM had its wind speed hourly values multiplied by a factor to match the cumulative frequency of the observational data daily mean values. For Abu Dhabi, data on atmospheric pressure could not be found; a static standard atmospheric pressure was used since the city is located at sea level. Note that observations of solar radiation were not available for Singapore, so its solar irradiance was not bias-corrected when the datasets were prepared.

**Table 5:** Observational data used in the bias-correction step for each city.

CZ	City	Latitude (°)	Longitude (°)	Observational data Period	Data used for bias correction					
					tas	hurs	rsds	sfcWind	ps	clt
0A	Singapore	1.37	103.98	1996–2015	x	x		x	x	
0B	Abu Dhabi	24.42	54.61	2008–2012	x	x	x	x	x	
1A	Guayaquil	−2.15	−79.92	07-2016 – 08-2020	x	x	x	x		
2A	Sao Paulo	−23.63	−46.65	1986–2005	x	x	x*	x*	x	
3A	Buenos Aires	−34.56	−58.42	1986–2005	x	x	x	x	x	
3A	Rome	41.81	12.25	2008–2017	x	x	x	x	x	
3B	Los Angeles	33.93	−118.40	2000–2019	x	x	x	x	x	
4A	Brussels	50.80	4.36	2009–2018	x	x	x	x		
4A	Ghent	51.05	3.73	2009–2020	x	x	x	x	x	
4A	London	51.48	−0.45	1996–2015	x	x	x	x	x	x
4C	Vancouver	49.19	−123.18	1998–2017	x	x	x	x	x	
5A	Toronto	43.67	−79.40	1998–2017	x	x	x	x	x	
5A	Copenhagen	55.88	12.41	2001–2019	x	x	x	x	x	
6A	Montreal	45.63	−73.55	1998–2017	x	x	x	x	x	
6A	Stockholm	59.9	18.03	1986–2005	x	x	x	x	x	x

x* = hourly values estimated from daily values.

The coordinates given in Table 5 correspond to the location of the weather station where the observations were used for bias correction for each city. The chosen weather stations are located outside of the cities, usually at airport sites; therefore, the observations and the resulting bias-corrected datasets do not account for urban heat island effects (UHI). We decided not to include urban effects in these datasets for various reasons. First, urban observations are not available for some of the cities analyzed. Secondly, the UHI is not homogeneous across a city, varying significantly depending on the different local climate zones (LCZ). Therefore, it would be necessary to create more than one urban weather file for each city, namely one for each LCZ. Furthermore, it would not be correct to use current urban observations as a reference for future UHI intensities because building density, vegetation, materials, and anthropogenic heat generation in future cities will probably change, leading to a change in UHI intensity. For all these reasons, even if the datasets refer to cities, they do not include urban effects, like most of the currently available weather datasets for building performance simulations. They can be modeled and added to the datasets in post-processing by using tools and methodologies that are discussed and referenced in the “Usage notes” section.

### Calculating direct and diffuse solar irradiance

The Boland–Ridley model^49^ was used to calculate the direct and diffuse components of global solar irradiance. This method is a robust and straightforward predictor model that requires few inputs. The Italian National organization for standardization (UNI) has adopted this reliable method to split the global solar irradiance for creating national climatic data (UNI 10349-1:2016)^50^. The model was also validated in a later study^51^. The Boland–Ridley model uses a logistic function (sigmoid function) for the diffuse fraction of global solar irradiance on a horizontal surface based on the sky clearness index, which is the ratio of the terrestrial global horizontal solar irradiance to the extraterrestrial horizontal solar irradiance. The extraterrestrial horizontal solar irradiance is calculated from the solar elevation and the extra-atmospheric solar irradiance received on a theoretical surface orthogonal to the sun’s rays and at the Earth’s mean distance from the sun (depending on the Earth’s orbital angle). This fraction includes both the horizontal direct and diffuse solar irradiance components of horizontal solar irradiance. This model is used for the generation of direct-normal solar irradiance^52^, which is required for building energy simulation. It is computed as the ratio of the direct horizontal solar irradiance to the cosine of the solar zenith angle. Calculation of direct-normal solar irradiance can yield unphysical results when the direct-horizontal solar irradiance and the cosine of the solar zenith angle are both small because the sun is low. In this case, a threshold is introduced by applying a physical model^53^ that considers the Rayleigh optical depth (in the function of the air mass) and the Linke Turbidity (TL)^54^, which accounts for scattering and absorption by both atmospheric aerosols and atmospheric gases.

### Creating typical years from multiyear hourly datasets

The TMYs were created using the international standard EN ISO 15927-4 – Hygrothermal performance of buildings, Calculation and presentation of climatic data, Part 4: Hourly data for assessing the annual energy use for heating and cooling method^5^. The procedure is applicable for assessing the climate change impact on the long-term mean energy loads of buildings. However, this method based on average values is not suitable for studying extreme meteorological events. TMYs are constructed from 12 representative months (typical months) from multiyear records. Two sets of parameters are considered for selecting the typical months: primary parameters, including dry-bulb air temperature, global solar irradiance, and relative humidity (or air absolute humidity, water vapor pressure, or dew point temperature), and secondary parameters, including wind speed. For each primary climatic parameter, *p*, the daily means, $\bar{p}$, are calculated from all multi-year records of hourly values of *p* (at least ten years). After sorting the $\bar{p}$ values for a specific month, *m*, of all the years in increasing order, the cumulative distribution function is calculated for each parameter and *i*^th^ day as:

1 $$\Phi \left(p,m,i\right)=\frac{K\left(i\right)}{N+1}$$

where *K*(i) is the rank order of the *i*^th^ day and *N* is the number of days for a month overall multi-year records. Afterward, the cumulative function is calculated for each year of the multi-year records for a specific month, *m*, and specific year, *y*, according to Eq. 2:

2 $$F\left(p,y,m,i\right)=\frac{J\left(i\right)}{n+1}$$

where *J*(i) is the rank order of the *i*^th^ day and *n* is the number of days for the specific month and year. Subsequently, the Finkelstein–Schafer statistic (F_s_)^55^ is calculated for all the primary climatic parameters for each calendar month and year of multi-year records. F_s_ is a goodness-to-fit statistic that proved more potent than conventional alternatives and is calculated as:

3 $${F}_{s}(p,y,m=\mathop{\sum }\limits_{i=1}^{n}| F(p,y,m,i)-\Phi (p,m,i)| $$

For each calendar month and each year, *Fs* values are calculated and ranked in increasing order. By calculating the total ranking (the sum of the primary parameter’s ranks) for each year, three months with the lowest total ranking are selected for each calendar month. The month with the lowest deviation in wind speed (secondary parameter) is selected as the typical month to be included in the typical year. This method was applied to the 20-year bias-corrected RCM data to generate one TMY for each period. The TMYs were then converted to EnergyPlus weather files (.EPW) for use in building energy simulations. The EnergyPlus auxiliary program “weather converter” tool^56^ was used for this purpose.

### Selecting extreme heatwaves from multi-year datasets

The method proposed by Ouzeau *et al*.^6^ was used to select heatwaves from the 20-year periods based on high quantiles of daily temperature distributions. The method was validated for France by comparing heatwave detection on an EURO-CORDEX regional multi-model ensemble with the French SAFRAN thermal indicator, historically used by French authorities for cold spell detection. The adopted method has the advantage of applying to different cities worldwide since it is based on relative thresholds and not absolute thresholds. It detects heatwaves based on three temperature thresholds calculated from the historical multiyear period: The 99.5 threshold (99.5 percentile) is used to detect a temperature peak and a potential heatwave. The 97.5 threshold (97.5 percentile) is used to calculate the heatwave duration (days during which the temperature is above the threshold) and severity (degree-days above the threshold). If the temperature goes under this threshold for more than three consecutive days, the heatwave stops. The 95 threshold (95 percentile) is used to end the heatwave drastically if the temperature drops below this threshold. The chosen method was recently demonstrated to be the most effective in detecting and characterizing heat waves for building resilience analysis^57^. The current work builds on the methodology initiated by Machard *et al*.^3^ to assemble future weather files, including heatwave for building energy and thermal performance simulations from CORDEX climate data. In the proposed approach, each heatwave is characterized by three criteria: intensity (maximum daily mean temperature °C reached during the heatwave), duration (in days), and severity (aggregated temperature above the 97.5 threshold in °C.day). Applying this method, many heatwaves were found during each multiyear period in each city. Since the purpose of the datasets is to carry out building performance resilience assessments, the three most extreme heatwaves were selected, according to these three criteria: the most intense, the most severe, and the longest heatwaves.

## Data Records

The entire datasets (Table 6) produced for this work are organized into three categories:

- Multiyear (MY)
- Typical meteorological year (TMY)
- Heatwave year (HWY)

**Table 6:** Datasets available for each city and data periods (Historical 2001 −2020, Mid-term future 2041–2060, Long-term Future (2081–2100).

Category	Short description	Extension	Link
MY - Multiyear dataset	A file containing hourly values of 20-years bias-corrected climate data	csv	https://www.wdc-climate.de/ui/entry?acronym=WDTF_Annex80_build_v1.0^58^
TMY - Typical Meteorological Year	Weather file to run building performance simulations representative of typical meteorological conditions over 20 years	epw	
HWY – Heatwave year (year containing heatwaves)	Weather file to run building performance simulations including extreme heatwaves (i.e., most severe, longest, or most intense over 20 years)	epw	

The datasets are available at the link: https://www.wdc-climate.de/ui/entry?acronym=WDTF_Annex80_build_v1.0^58^.

The first category of files is MY datasets in CSV format. There are three MY files for each city, containing the hourly values of the bias-corrected RCM variables for each 20-year reference period. The variables included in the CSV files are air temperature (tas), near-surface relative humidity (hurs), near-surface specific humidity (huss), surface atmospheric pressure (ps), surface downwelling shortwave irradiance (rsds), and wind speed (sfcWind). Some cities have fewer variables due to missing observational data to perform the bias-correction. Cloud cover (clt) is available for London and Stockholm. The MY file name format is: “climatezone_city_MY_referenceperiod.csv”. For instance: “0B_Abu Dhabi_MY_2081–2100”. The MY files were used to create both TMYs and HWYs.

There are three TMYs per city, representing the typical meteorological conditions corresponding to historical (2001–2020), mid-term future (2041–2060), and long-term future (2081–2100) periods. The TMYs are provided in the EnergyPlus weather file (EPW) format. The EPW file details hourly dry bulb air temperature (°C), dew point temperature (°C), relative humidity (%), atmospheric pressure (Pa), global horizontal solar irradiance (Wh/m^2^), direct normal irradiance (Wh/m^2^), diffuse horizontal irradiance (Wh/m^2^), wind speed (m/s), and wind direction (°). For the cities of London and Stockholm, the total sky cover (tenths) is also provided. In TMYs, values for wind direction were extracted from the historical time series of METEONORM^59^ for each city because wind direction is needed to perform building energy simulations but is not available for all CORDEX domains. The EPW files were generated using the EnergyPlus weather converter, auxiliary software of EnergyPlus^56^.

The file name of each TMY has the following format: “climatezone_city_TMY_referenceperiod.epw”. For instance, the file “4 A_London_TMY_2041–2060” is the TMY for the city of London, located in the ASHRAE climate zone 4A,for the mid-term future period (2041- 2060).

Finally, the HWYs are also provided in EPW format. Each city can have a maximum of nine HWY files, corresponding to the years with the most intense, most severe, and longest heatwaves found in the three reference periods. As the most intense and/or the longest heatwaves are also the most severe in many cases, the total number of HWY files is generally less than nine. The HWY file name format is “climatezone_city_HW_referenceperiod_heatwavetype_year.epw”. For instance, the file “6 A_Stockholm_HW_Historical_MostSevere_Longest_2002.epw” contains the most severe and longest heatwave occurring in the historical period, in 2002, in Stockholm (climate zone 6A).

## Technical Validation

For technical validation, the multiyear raw climate outputs, observations, and bias-adjusted datasets were compared and analyzed. The mean values of ambient air temperature, relative humidity, global solar irradiance, and wind speed in the typical years during the historical period were compared to the mean values in the multiyear datasets, showing good agreement in values. The extreme values of ambient air temperature for the heatwave years were compared to the extreme of the multiyear datasets. An assessment of the future weather files confirms that climate change will increase the mean temperature in all cities. Heatwave frequency, intensity, and duration will also increase in all cities and more drastically in the four hottest cities (Singapore, Abu Dhabi, Guayaquil, and Sao Paulo) analyzed.

### Comparison of raw-output and bias-corrected data

**Fig. 4:**
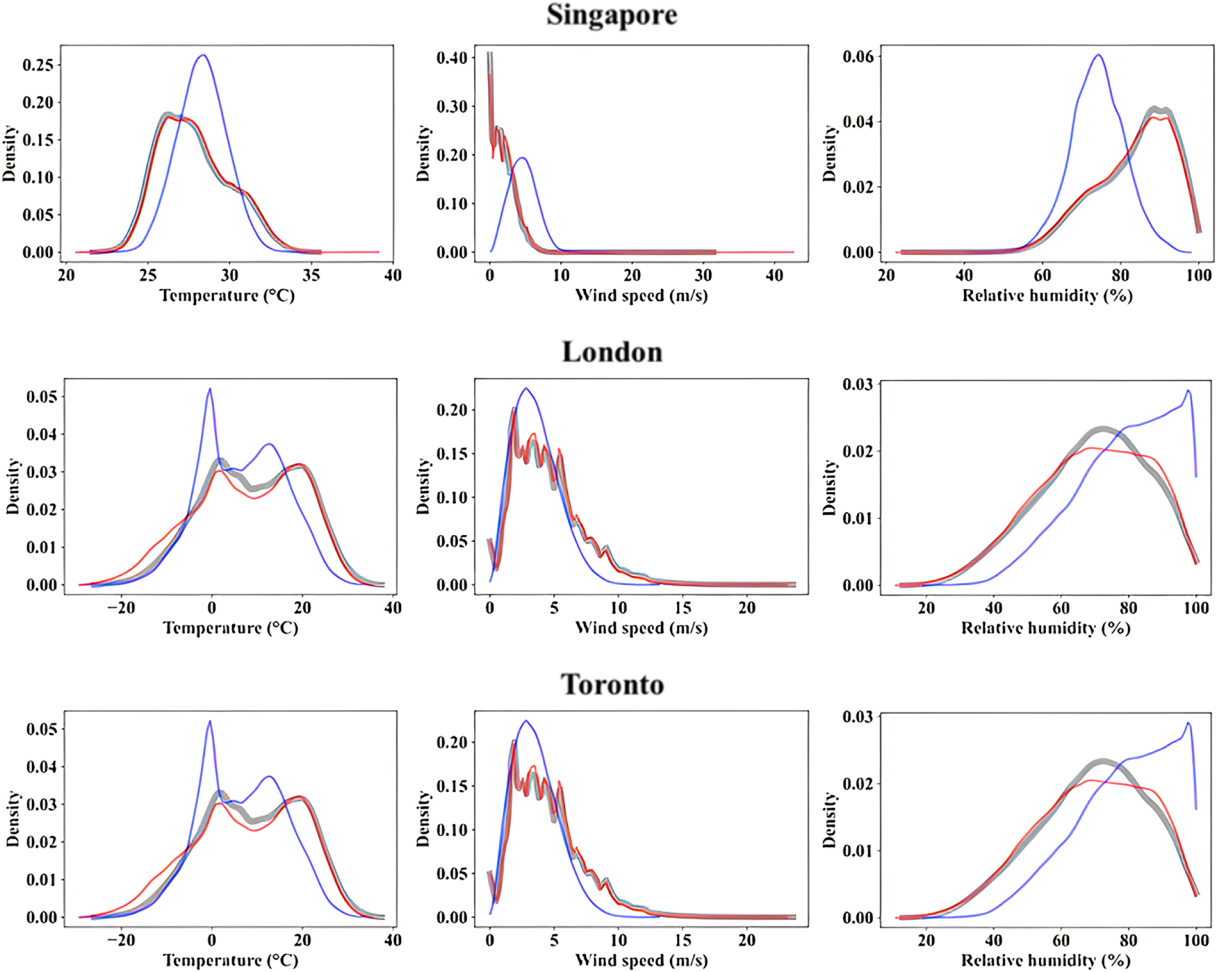
Probability density functions of temperature, wind speed, and relative humidity in Singapore, London, and Toronto from observations (grey), raw RCM (blue), and bias-corrected RCM (red) datasets over the validation time period.

The validation of the bias-correction step was performed by comparing bias-corrected climate estimates with observations over a validation time-period that varies from city to city depending on the time period of observations available to them. The validation time period is considered the period overlapping between observational and historical time-periods. This allowed us to make use of the entire length of observational data available in different cities for performing validation of bias-correction methods. The validation results show that the QDM/MBCn methods were able to reduce the bias associated with RCM simulations effectively. This can be seen from the results presented in Table 7, in which mean climate statistics from observations, raw RCM, and bias-corrected (bc) RCM are presented for the validation time period. The results show that the projected temperature, solar irradiance, wind speed, and relative humidity from raw RCMs have noticeable bias, which is reduced by the application of the bias-correction step. For instance, RCM over-predicts the mean temperature in Singapore by 0.5 °C, which is effectively eliminated after the bias correction. Table 8 presents the standard deviation of observations (OBS), RCM-raw, and RCM-bs for these four climate variables, which also shows the bias reduction between OBS and bias-corrected RCM data. Not only is the bias correction effective in correcting bias in average climate characteristics over the cities, but it also reduces bias across the whole distribution of climate variables. This is evident from Fig. 4, in which probability density functions (PDFs) of temperature, wind speed, and relative humidity from observations (grey), raw RCM (blue), and bias-corrected RCM (red) datasets are presented for Singapore, London, and Toronto. PDFs of raw RCM are effectively adjusted by the bias-correction procedure to mimic the PDFs of observations. This is true not only for temperature but also for relatively more complex variables such as wind speed, highlighting the effectiveness of the bias-correction step in simulating realistic estimates of a range of climate variables considered in this study.

**Table 7:** Mean temperature, solar irradiance, wind speed, and relative humidity in the cities over the validation time period.

CZ	City	Temperature (°C)			Solar irradiance (W/m^2^)			Wind speed (m/s)			Relative humidity (%)		
		*OBS*	*RCM (raw)*	*RCM (bc)*	*OBS*	*RCM (raw)*	*RCM (bc)*	*OBS*	*RCM (raw)*	*RCM (bc)*	*OBS*	*RCM (raw)*	*RCM (bc)*
0A	Singapore	27.8	28.1	27.7	—	—	—	1.9	4.7	2.0	83.8	74.1	83.3
0B	Abu Dhabi	27.6	29.0	27.6	237.9	246.7	238.1	3.2	4.0	3.2	60.0	55.5	60.2
1A	Guayaquil	27.0	27.1	27.0	263.1	218.4	266.9	1.7	1.9	1.8	74.6	77.4	74.3
2A	Sao Paulo	19.3	19.4	19.3	188.6	187.9	188.6	6.1	2.73	6.1	80.6	77.1	80.6
3A	Buenos Aires	18.0	19.1	17.7	191.4	197.5	190.0	4.5	4.5	4.4	72.1	66.9	72.3
3A	Rome	16.3	16.5	16.3	187.8	163.9	188.0	3.6	2.7	3.6	72.5	70.0	72.4
3B	Los Angeles	16.7	20.7	16.7	215.1	223.3	214.7	1.7	2.4	1.7	72.3	57.5	72.4
4A	Brussels	10.8	11.1	10.8	127.2	109.1	127.2	3.6	3.7	3.6	78.5	82.0	78.5
4A	Ghent	11.1	11.3	11.1	126.4	110.3	126.3	3.4	4.1	3.4	78.6	82.8	78.7
4A	London	11.6	11.1	11.8	118.8	106.8	117.5	4.2	2.8	4.0	75.5	79.5	75.7
4C	Vancouver	10.1	7.8	10.6	142.7	130.5	153.7	3.7	3.3	4.4	78.9	69.2	74.5
5A	Toronto	9.2	6.6	7.8	159.0	146.6	153.7	4.4	3.6	4.4	69.3	79.1	69.2
5A	Copenhagen	8.8	9.3	8.8	118.2	102.1	118.2	3.3	4.6	3.3	82.4	84.9	82.4
6A	Montreal	7.7	4.9	7.8	153.8	134.8	153.7	4.4	3.5	4.4	69.2	83.6	69.2
6A	Stockholm	6.6	6.4	6.6	116.5	92.9	116.6	3.9	3.0	3.9	79.6	86.0	79.6

**Table 8:** Standard deviation of temperature, solar irradiance, wind speed, and relative humidity in the cities over the validation time period.

CZ	City	Temperature (°C)			Solar irradiance (W/m^2^)			Wind speed (m/s)			Relative humidity (%)		
		*OBS*	*RCM (raw)*	*RCM (bc)*	*OBS*	*RCM (raw)*	*RCM (bc)*	*OBS*	*RCM (raw)*	*RCM (bc)*	*OBS*	*RCM (raw)*	*RCM (bc)*
0A	Singapore	2.2	1.5	2.1	—	—	—	1.6	1.9	1.6	9.9	6.9	10.0
0B	Abu Dhabi	7.9	7.7	7.9	312.4	326.9	313.2	2.2	2.1	2.2	20.4	21.4	20.5
1A	Guayaquil	3.4	3.3	3.4	380.8	312.1	382.3	1.0	0.9	1.0	12.4	14.4	12.0
2A	Sao Paulo	4.7	4.6	4.7	256.4	288.3	256.4	2.9	1.3	3.0	16.4	16.6	16.4
3A	Buenos Aires	5.6	5.1	5.7	283.5	297.3	282.5	2.4	1.9	2.4	15.2	16.3	15.4
3A	Rome	7.1	7.3	7.1	270.4	255.6	270.4	2.2	1.7	2.2	16.7	17.6	16.7
3B	Los Angeles	4.4	7.4	4.4	295.9	310.6	296.1	1.0	1.3	1.0	22.3	22.8	22.2
4A	Brussels	6.8	6.8	6.8	196.2	201.0	196.1	1.8	1.8	1.8	14.3	14.1	14.3
4A	Ghent	6.8	6.6	6.8	201.0	193.5	200.6	1.9	2.0	1.9	15.4	13.5	15.2
4A	London	6.1	6.3	6.1	193.1	195.9	191.3	2.2	1.2	2.2	15.9	14.0	15.7
4C	Vancouver	5.3	12.0	6.0	231.2	511.1	235.4	2.3	2.2	2.5	13.0	16.8	12.7
5A	Toronto	10.9	9.2	12.0	241.4	222.8	235.4	2.7	1.8	2.5	16.2	14.5	16.8
5A	Copenhagen	7.2	6.3	7.2	196.0	191.4	196.1	2.1	2.2	2.1	15.4	11.7	15.4
6A	Montreal	12.0	8.7	12.0	235.4	213.4	253.4	2.6	1.8	2.5	16.8	13.4	16.8
6A	Stockholm	7.9	7.7	7.9	184.7	178.0	184.7	1.7	1.4	1.7	14.6	11.7	14.6

### Projected changes in weather variables over multi-year (MY) future periods

The values of mean temperature, solar irradiance, wind speed, and relative humidity over the 2010s, 2050 s, and 2090 s for all cities are presented in Table 9. In general, between the historical period (the 2010s) and the two future time periods (2050 s, 2090 s), mean temperatures are projected to increase in all cities located in different climate zones (CZs). In most cities, the increase in MY by the 2050 s is about 1 °C, while it will be about 2-3 °C by the 2090 s, with the largest increase of 4.2 °C in Abu Dhabi (CZ: 0B – Extremely Hot Dry) and the smallest increase of 1.6 °C in Buenos Aires (CZ: Warm Humid). Mean temperature increases within the same ASHRAE climate zone are consistent: in zone ASHRAE CZ: 4 A - Mixed Humid, the temperature increase in Brussels, Ghent, and London are about 0.8 °C, 0.7 °C, and 0.7 °C between MY-2050s and MY-2010s, and of 2.6 °C, 2.6 °C, and 2.5 °C between MY-2090s and MY-2010s. Global solar irradiance is projected to decrease in the majority of the cities, with the largest decrease of 12.8 W/m^2^ by the 2090 s is projected for Stockholm (CZ: Cold Humid), whereas a slight increase of 0.6 W/m^2^ is projected for Abu Dhabi (CZ 0B -: Extremely Hot Dry). Such a reduction in future solar irradiance was also found in other studies^45,60^. According to Cutforth and Judiesch^61^, this can be the consequence of two factors: (1) higher attenuation of solar irradiance from increased aerosol concentrations and sometimes from increasing cloudiness, and (2) an increase in annual number of precipitation events. These assumptions are coherent since the irradiance is not decreasing in Abu Dhabi, for which cloud cover is very low. However, this trend in decreasing global solar irradiance cannot be generalized. It can be due to a coarse representation of rain and cloud events at the model spatial resolution (25 or 50 km depending on the CORDEX domain) and to potential biases for this climate parameter in the selected climate model. In terms of wind speed and relative humidity, a general change is not observed. Most cities have minimal change in wind projections in the future: The largest decrease of 0.4 m/s in wind speed is projected for Buenos Aires (CZ: Warm Humid), whereas the largest increase of 0.3 m/s in wind speed is projected for Sao Paulo (CZ: Hot Humid). Finally, the largest variability in the sign of projected future change is obtained for relative humidity. While the cities of Singapore (CZ: 0 A - Extremely Hot Humid), Guayaquil (CZ: 1 A - Very Hot Humid), Buenos Aires (CZ: 3 A - Warm Humid), Los Angeles (CZ: 3B - Warm Dry) are projected to experience increases in relative humidity of up to 5%, the cities of Sao Paulo (CZ: 2 A - Hot Humid) and Abu Dhabi (CZ: 0B - Extremely Hot Dry) are projected to experience future decreases of up to 4%. Smaller future changes in relative humidity are projected for other cities such as Montreal and Stockholm (CZ: 6 A - Cold Humid) as well as Ghent, Brussels, and London (CZ: 4 A - Mixed Humid and 5 A – Cold Humid).

**Table 9:** 20-year mean temperatures, solar irradiance, wind speed, and relative humidity in the cities over the 2010s, 2050 s, and 2090 s time periods obtained from multi-year bias-corrected RCM data.

CZ	City	Temperature (°C)			Solar irradiance (W/m^2^)			Wind speed (m/s)			Relative humidity (%)		
		*2010s*	*2050s*	*2090s*	*2010s*	*2050s*	*2090s*	*201s0s*	*2050s*	*2090s*	*2010s*	*2050s*	*2090s*
0A	Singapore	27.9	29.1 (1.2)	30.3 (2.4)	168.6	169.0 (0.4)	166.2 (−2.4)	2.0	2.0 (0.0%)	2.0 (0.0%)	83.2	84.7 (1.5)	84.6 (1.4)
0B	Abu Dhabi	27.7	29.3 (1.6)	31.9 (4.2)	240.3	241.1 (0.8)	240.9 (0.6)	3.2	3.2 (0.0%)	3.1 (−3.1%)	60.0	58.8 (−1.2)	57.3 (−2.7)
1A	Guayaquil	26.9	28.1 (1.2)	30.2 (3.3)	263.0	258.1 (−4.9)	253.5 (−9.5)	1.7	1.7 (0.0%)	1.7 (0.0%)	74.9	75.2 (0.3)	75.7 (0.8)
2A	Sao Paulo	19.8	21.3 (1.5)	23.3 (3.5)	188.8	188.5 (−0.3)	182.8 (−6.0)	6.1	6.0 (−1.6%)	6.0 (−1.6%)	80.3	80.0 (−0.3)	80.3 (0.0)
3A	Buenos Aires	17.9	18.7 (0.8)	19.5 (1.6)	188.4	184.5 (−3.9)	178.8 (−9.6)	4.3	4.2 (−2.3%)	3.9 (−9.3%)	73.5	75.7 (2.2)	78.3 (4.8)
3A	Rome	16.1	17.1 (1.0)	19.6 (2.5)	187.7	183.7 (−4.0)	185.7 (−2.0)	3.6	3.6 (0.0%)	3.5 (−2.8%)	72.1	73.3 (1.2)	71.4 (−0.7)
3B	Los Angeles	16.7	17.9 (1.2)	19.4 (2.7)	214.7	211.2 (−3.5)	205.9 (−8.8)	1.7	1.6 (−5.9%)	1.6 (−5.9%)	72.4	74.6 (2.2)	77.1 (4.7)
4A	Brussels	10.8	11.6 (0.8)	13.4 (2.6)	126.2	123.3 (−2.9)	118.3 (−7.9)	3.6	3.6 (0.0%)	3.6 (0.0%)	78.6	78.6 (0.0)	78.7 (0.1)
4A	Ghent	11.0	11.7 (0.7)	13.6 (2.6)	108.1	105.9 (−2.2)	101.7 (−6.4)	4.2	4.2 (0.0%)	4.2 (0.0%)	83.1	83.1 (0.0)	83.1 (0.0)
4A	London	12.0	12.7 (0.7)	14.5 (2.5)	118.4	115.2 (−3.2)	113.1 (−5.3)	4.0	4.0 (0.0%)	4.0 (0.0%)	75.1	75.3 (0.2)	74.9 (−0.2)
4C	Vancouver	7.8	9.1 (1.3)	10.9 (3.1)	153.8	149.4 (−4.4)	142.9 (−10.9)	4.4	4.3 (−2.3%)	4.1 (−6.8%)	69.2	69.5 (0.3)	70.4 (1.2)
5A	Toronto	7.9	8.9 (1.0)	11.1 (3.2)	153.7	153.4 (−0.3)	149.6 (−4.1)	4.4	4.3 (−2.3%)	4.2 (−4.6%)	68.9	68.7 (−0.2)	69.2 (0.3)
5A	Copenhagen	8.8	9.7 (0.9)	11.3 (2.5)	117.8	114.9 (−2.9)	108.0 (−9.8)	3.3	3.2 (−3.0%)	3.3 (0.0%)	82.4	82.6 (0.2)	83.0 (0.6)
6A	Montreal	7.9	8.9 (1.0)	10.9 (3.0)	154.3	156.8 (2.5)	152.0 (−2.3)	4.4	4.4 (0.0%)	4.2 (−4.6%)	69.0	68.6 (−0.4)	69.0 (0.0)
6A	Stockholm	7.7	8.9 (1.2)	10.6 (2.9)	116.4	110.5 (−5.9)	103.6 (−12.8)	3.8	4.0 (5.3%)	4.0 (5.3%)	79.1	78.9 (−0.2)	79.1 (0.0)

Cells with future projected increases (decreases) in climate variables are highlighted in red (green). Grey color means no change. Values in brackets represent the change (absolute value for temperature, solar irradiance, and relative humidity, relative change for wind speed) between the selected term and the 2010s.

**Fig. 5:**
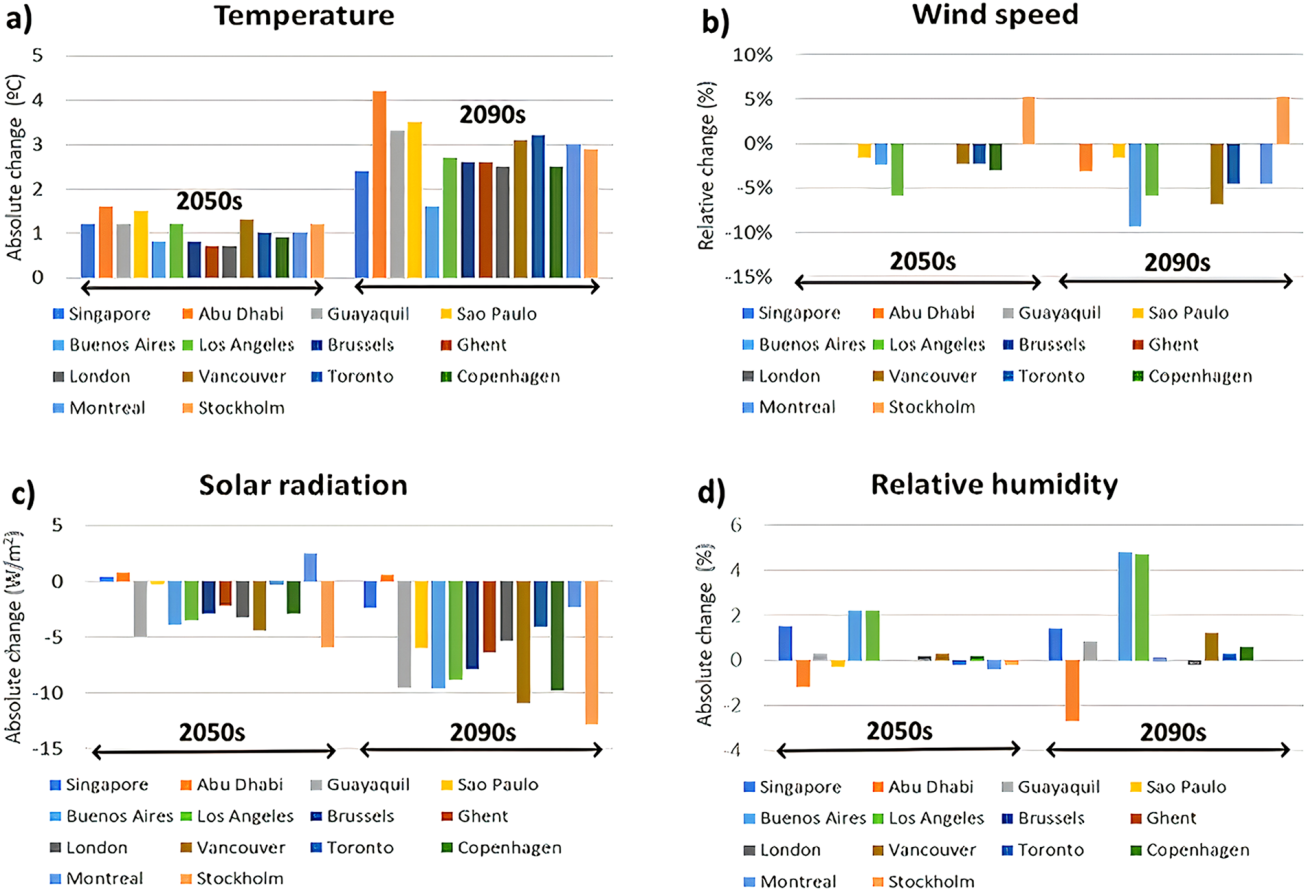
Changes in climatic variables from the 2010s to 2050 s and 2090 s: (**a**) absolute change for temperature, (**b**) relative change in wind speed, (**c**) absolute change in solar radiation, (**d**) absolute change in relative humidity.

The change between future 20-year periods (2050 s and 2090 s) compared to the present period (2010s) in presented for the mean temperature, mean solar irradiance, mean wind speed, and mean relative humidity in Fig. 5.

Table 10 highlights changes at the 99 percentiles of the multi-year distributions. A sharp increase in temperatures is witnessed, especially in the four hottest cities, with changes up to + 5.8 °C by the end of the century (i.e., Sao Paulo). For the solar irradiance, wind speed, and relative humidity, similar trends are observed for the mean values.

**Table 10:** 20-year 99% temperatures, solar irradiance, wind speed, and relative humidity in the cities over the 2010s, 2050 s, and 2090 s time periods obtained from multi-year bias-corrected RCM data.

CZ	City	Temperature (°C)			Solar irradiance (W/m^2^)			Wind speed (m/s)			Relative humidity (%)		
		*2010s*	*2050 s*	*2090 s*	*2010s*	*2050 s*	*2090 s*	*2010s*	*2050 s*	*2090 s*	*2010s*	*2050 s*	*2090 s*
0A	Singapore	33.0	34.4 (1.4)	38.2 (5.2)	965.1	960.0 (−5.1)	949.4 (−15.7)	6.2	6.2 (0.0%)	6.0 (−3.3%)	99.4	100 (0.6)	100 (0.6)
0B	Abu Dhabi	44.3	46.4 (2.1)	49.5 (5.2)	940.3	934.9 (−5.4)	927.3 (−13.0)	9.2	9.1 (−1.1%)	9.0 (−2.2%)	95.1	94.8 (−0.3)	94.4 (−0.7)
1A	Guayaquil	34.6	35.7 (1.1)	37.8 (3.2)	1,296.5	1,281.0 (−15.5)	1,260.3 (−36.2)	4.7	4.7 (0.0%)	4.5 (−4.4%)	99.0	99.0 (0.0)	99.0 (0.0)
2A	Sao Paulo	31.0	33.6 (2.6)	36.8 (5.8)	895.6	891.4 (−4.2)	881.7 (−13.9)	13.9	14.1 (1.4%)	14.5 (4.1%)	100	100 (0.0)	97.7 (−2.3)
3A	Buenos Aires	29.5	30.0 (0.5)	30.6 (1.1)	1,011.0	1,002.8 (−8.2)	994.7 (−16.3)	11.1	10.9 (−1.8%)	10.4 (−6.7%)	100	100 (0.0)	100 (0.0)
3A	Rome	30.6	32.0 (1.4)	35.9 (5.3)	906.0	898.8 (−9.2)	892.0 −14)	10.5	10.5 (0.0%)	10.4 (−1.0%)	100	100 (0.0)	100 (0.0)
3B	Los Angeles	28.1	29.8 (1.7)	31.0 (2.9)	956.0	946.1 (−9.9)	934.9 (−21.1)	4.2	4.2 (0.0%)	4.2 (0.0%)	100	100 (0.0)	100 (0.0)
4A	Brussels	26.4	28.0 (1.6)	29.3 (2.9)	759.0	749.7 (−9.3)	733.1 (−25.9)	8.7	9.0 (3.3%)	9.0 (3.3%)	98.9	98.8 (−0.1)	98.8 (−0.1)
4A	Ghent	26.9	28.1 (1.2)	29.5 (2.6)	779.4	771.6 (−7.8)	753.8 (−25.6)	9.6	9.8 (2.0%)	9.9 (3.0%)	100	100 (0.0)	100 (0.0)
4A	London	26.7	27.8 (1.1)	29.9 (3.2)	779.4	770.6 (−8.8)	763.6 (−15.8)	10.5	10.7 (1.9%)	10.6 (0.9%)	98.0	98.0 (0.0)	98.0 (0.0)
4C	Vancouver	28.9	30.4 (1.5)	31.7 (2.8)	882.5	872.7 (−9.8)	858.6 (−23.9)	11.8	11.6 (−1.7%)	11.5 (−2.6%)	98.3	98.1(−0.2)	98.1(−0.2)
5A	Toronto	29.0	30.7(1.7)	32.9(3.9)	882.4	879.6(−2.8)	863.6(−18.8)	11.9	11.7(−1.7%)	11.6(−2.6%)	98.1	98.2(0.1)	98.2(0.1)
5A	Copenhagen	24.7	25.7(1.0)	26.4(1.7)	779.1	770.9(−8.2)	757.8(−21.3)	9.2	9.2(0.0%)	9.3(1.1%)	100	100(0.0)	100(0.0)
6A	Montreal	29.0	30.0(1.0)	31.8(2.8)	883.6	881.0(−2.6)	872.8(−10.8)	11.9	11.7(−1.7%)	11.4(−4.4%)	97.8	97.9(0.1)	97.3(−0.5)
6A	Stockholm	24.2	24.9(0.7)	25.6(1.4)	699.0	696.3(−2.7)	682.7(−16.3)	8.4	8.7(3.4%)	8.8(4.5%)	99.0	99.0(0.0)	98.9(−0.1)

Cells with future projected increases (decreases) in climate variables are highlighted in red (green). Grey color means no change.

### Projected changes in weather variables of typical meteorological years (TMY) for building performance simulations

Table 11 presents the values of mean temperatures, solar irradiance, wind speed, and relative humidity in the three typical meteorological years (TMY) generated from each 20-year dataset. The projected changes in climate variables in the future TMYs are generally consistent with those resulting from the comparison of the 20-year datasets. This means that the TMYs are indeed representative of the climate projections over an interval (i.e., 20 years) and thus suitable for assessing the impact of climate change on building energy loads.

**Table 11:** Mean temperatures, solar irradiance, wind speed, and relative humidity in the three TMYs weather files generated based on the bias-corrected 20-years datasets for each city.

CZ	City	Temperature (°C)			Solar irradiance (W/m^2^)			Wind speed (m/s)			Relative humidity (%)		
		*2010s*	*2050 s*	*2090 s*	*2010s*	*2050 s*	*2090 s*	*2010s*	*2050 s*	*2090 s*	*2010s*	*2050 s*	*2090 s*
0A	Singapore	27.9	29.1	30.3	163.7	167.5	165.6	2.1	2.0	2.0	82.7	84.8	84.8
0B	Abu Dhabi	27.9	29.4	31.7	233.9	235.5	234.7	3.2	3.2	3.1	59.7	58.4	57.7
1A	Guayaquil	27.1	28.5	30.4	255.5	243.4	232.5	1.7	1.8	1.6	73.4	71.8	75.8
2A	Sao Paulo	19.8	21.3	23.1	190.9	193.3	183.8	6.0	6.0	6.0	80.3	80.6	80.6
3A	Buenos Aires	17.8	18.9	19.5	192.8	190.1	182.9	4.3	4.1	3.9	73.5	74.9	78.5
3A	Rome	16.1	17.2	19.5	189.4	185.6	187.2	3.6	3.6	3.6	73.2	72.5	70.5
3B	Los Angeles	16.7	17.8	19.3	219.8	210.1	206.1	1.7	1.6	1.6	71.4	75.5	79.0
4A	Brussels	11.2	11.4	13.5	124.9	119.9	118.8	3.7	3.7	3.7	79.5	78.6	78.4
4A	Ghent	10.9	11.5	13.3	124.5	119.0	118.5	3.4	3.5	3.4	79.0	79.6	79.2
4A	London	12.1	12.7	14.2	116.9	114.4	110.6	4.1	4.0	4.0	75.1	75.2	76.0
4C	Vancouver	7.7	9.3	10.7	156.3	147.2	147.8	4.7	4.2	4.0	67.6	71.5	76.0
5A	Toronto	8.2	9.3	11.4	155.1	155.4	149.8	4.3	4.4	4.2	71.0	67.4	69.5
5A	Copenhagen	9.0	9.7	11.2	119.8	113.1	107.4	3.3	3.2	3.4	81.2	83.3	83.0
6A	Montreal	7.9	9.0	10.8	153.9	156.5	149.0	4.3	4.7	4.4	69.8	70.1	69.9
6A	Stockholm	7.9	8.9	10.7	119.5	111.9	108.8	3.8	3.9	4.0	79.7	79.7	78.4

The air temperature is consistently higher in the future weather files for all the cities, with a higher increase in the long-term (2090 s) future TMY than in the mid-term (2050 s) future TMY. The 2090s-TMY of Abu Dhabi (CZ: 0B Extremely Hot Dry) has the largest increase in temperature of 3.8 °C whereas the TMY of Buenos Aires (CZ: 3 A Warm Humid) has the smallest increase of 1.7 °C for the same period. Many cities are projected to have significantly higher increases in temperature in the long-term than in the mid-term (e.g., Brussels, Ghent, and London). These results are in close agreement with the changes obtained from the 20-year projections. As for the MYs, global solar irradiance will be reduced in the future TMYs of most cities. This is also in agreement with the 20-year projections. The 2090-TMY of Guayaquil (CZ: 1 A Very Hot Humid) has the largest decrease in solar irradiance (23.0 W/m^2^). The TMYs with slight increases in long-term global solar irradiance are those of Singapore (CZ: 0 A Extremely Hot Humid) and Abu Dhabi (CZ: 0B Extremely Hot Dry). Regarding wind speed, the changes between the 2010s, 2050 s, and 2090 s weather files are minimal. The 2090s-TMY of Vancouver (CZ: 4 C Mixed Marine) has the largest decrease in mean wind speeds of 0.7 m/s.

Finally, the future TMYs reflect a high variability in the sign of future changes in relative humidity in agreement with the results of the 20-years projections. The cities of Singapore (CZ: 0 A Extremely Hot Humid), Guayaquil (CZ: 1 A Very Hot Humid), Buenos Aires (CZ: 3 A Warm Humid), Los Angeles (CZ: 3B Warm Dry) and Vancouver (CZ: 4 C Mixed Marine) have an absolute increase in relative humidity up to 8% in the 2090-TMYs while Abu Dhabi (CZ: 0B Extremely Hot Dry) has a reduction of relative humidity in the 2090-TMY of 2%. The other cities have relatively smaller changes in relative humidity in future TMYs. This variability can be explained by two phenomena. On the one hand, there is general warming, and warmer air can hold more water vapor (air can contain about 7% more moisture for every 1 °C temperature increase according to the Clausius-Clapeyron equation). On the other hand, global warming leads to more evaporation of water and, thus, an increase in specific humidity. Therefore, to keep relative humidity the same, specific humidity must also increase by 7% per °C of warming. However, the oceans are warming more slowly than the land surface, which also means that not enough moisture has evaporated, and relative humidity has, therefore, been reduced.

### Projected changes in heatwaves (HWY) and selected extreme heatwaves for building performance simulations

Table 12 presents the three thresholds calculated for each city from the 20-year bias-adjusted historical daily temperatures data from 2001 to 2020 for heatwave selection. The relative thresholds are similar for all cities, resulting in different absolute thresholds presented in Table 12. Abu Dhabi is the city with the highest daily mean temperatures. The three European cities in CZ 4 A have equivalent thresholds. For the colder climate zones 5 A and 6 A, Toronto and Montreal in the eastern of Canada have similar thresholds, while European cities Copenhagen and Stockholm also have similar thresholds.

**Table 12:** Thresholds used over the historical period 2010s (2001–2020) for heatwave selection and number of heatwaves found per period in each city.

CZ	City	CORDEX Domain	Threshold to detect heatwaves over 2010s			Number of heatwaves detected		
			95 Threshold (°C)	97.5 Threshold (°C)	99.5 Threshold (°C)	2010s	2050 s	2090 s
0A	Singapore	SEA	30.4	30.9	31.7	7	58	136
0B	Abu Dhabi	SEA	37.1	38.1	39.3	5	47	61
1A	Guayaquil	AFR	29.3	29.8	30.8	8	40	207
2A	Sao Paulo	SAM	25.3	26.3	28.3	7	87	172
3A	Buenos Aires	SAM	25.3	26.4	28.0	6	19	32
3A	Rome	EUR	25.6	26.4	27.9	7	21	36
3B	Los Angeles	NAM	22.0	22.8	24.4	3	40	81
4A	London	EUR	20.6	21.9	24.3	6	16	46
4A	Brussels	EUR	20.4	22.0	24.6	9	14	36
4A	Ghent	EUR	20.2	21.8	25.0	7	20	33
4C	Vancouver	NAM	24.0	25.3	27.5	8	23	54
5A	Toronto	NAM	23.3	24.2	25.7	4	39	85
5A	Copenhagen	EUR	18.7	20.1	22.3	10	19	23
6A	Montreal	NAM	23.3	24.1	25.5	4	38	88
6A	Stockholm	EUR	18.6	19.8	21.7	9	25	27

Table 12 also presents the evolution in the number of heatwaves found during each 20-year period. While between 3 and 10 heatwaves are found during the historical period, depending on the cities, a substantial increase in heatwave numbers in the future will be observed in all cities. By 2050, the increase is more pronounced in cities in the four hottest climate zones, followed by cities in North America and then in Europe. Still, in the twenty-year period, every city displays at least one heatwave per summer on average by the mid-century. By the end of the century, the three cities in hot-humid climate zones (Singapore, Guayaquil, and Sao Paulo) showcase an impressive number of heatwaves, beyond a hundred, which would be equivalent to an average of five heatwaves per summer. In these cities, due to the large increase in temperatures, the heatwaves thresholds are exceeded many times during the same summer.

**Fig. 6:**
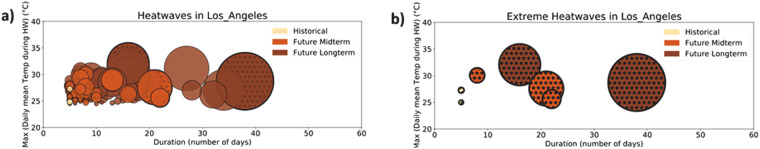
Heatwaves in Los Angeles (CZ 3B): (**a**) All heatwaves detected and (**b**) extreme heatwaves selection.

An illustration of the selection of the extreme heatwaves (the most intense, the most severe, and the longest of each period) is made in Fig. 6 for the city of Los Angeles. A bubble represents a heatwave, which size is linked to its severity. Figure 6a illustrates well the tremendous increase throughout the century and the diversity of heatwaves that are found as well. In comparison with the historical period, during which only very short heatwaves of five days are witnessed, in the mid-term future, longer heatwaves that are both less or more intense than the most intense heatwave of the historical period are found. By the end of the century, heatwaves are more severe and also longer. In Fig. 6b, the three most extreme heatwaves, the ones that are selected for future periods, are highlighted. During the 2050 s:

- the most intense heatwave is 8 days long with an intensity of 30.1 °C and a severity of 14.2 °C.d;
- the most severe heatwave is 21 days long with an intensity of 27.6 °C and a severity of 32.4 °C.d;
- the longest heatwave is 22 days long with an intensity of 25.6 °C and a severity of 17.5 °C.d;
  During the 2100 s, only two extreme heatwaves are selected:
- the most intense, which is also the most severe: intensity of 32.1 °C, duration of 16 days, and severity of 39.1 °C.d;
- the longest, which is 38 days long with an intensity of 28.7 °C and a severity of 53.6 °C.d

**Fig. 7:**
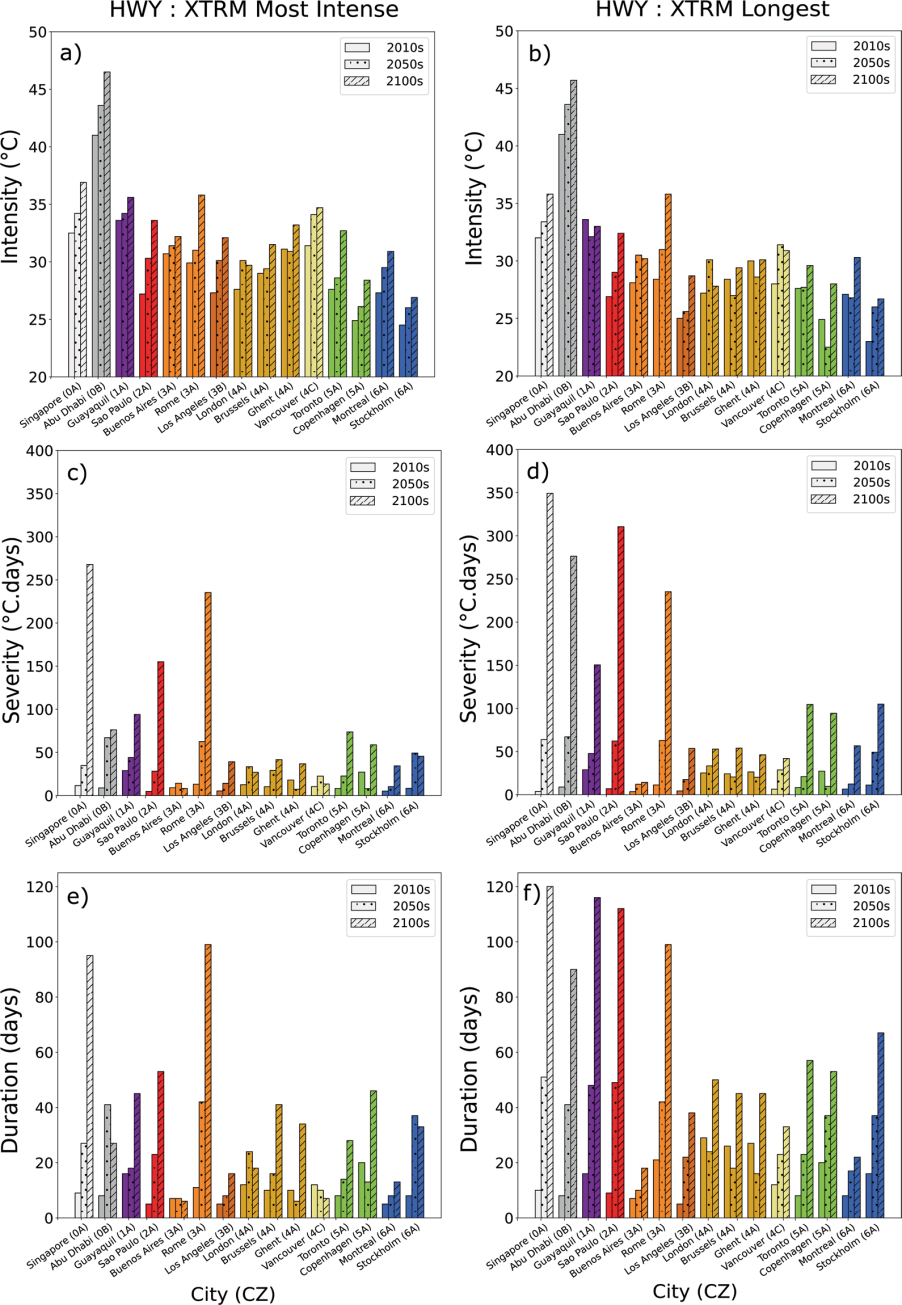
Characteristics (intensity, severity, and duration) of the most intense and longest XTRM-HW: (**a**) intensity of the most intense HW, (**b**) intensity of the longest HW, (**c**) severity of the most intense HW, (**d**) severity of the longest HW, (**e**) duration of the most intense HW, (**f**) duration of the longest HW.

For each city, the three extreme heatwaves (the most intense, most severe, and longest heatwave) are selected. Figure 7 shows the characteristics (intensity, severity, and duration) of the most intense and longest heat waves in each climate zone. Characteristics of the most severe heatwaves are often similar to the longest heatwaves and are not shown here.

The intensity of both extreme heatwaves strongly increases between the three periods and in each climate zone. The increase in intensity of the most intense heatwave by the end of the century is, in each city, between +2 °C (European cities in climate zone 4 A) and + 7 °C (Sao Paulo). The intensity of the longest heatwaves is between 0 °C and 3.4 °C (Vancouver), inferior to the most intense heatwaves in the 2010s, of 0 °C and 4.5 °C (Los Angeles) inferior in the 2050 s, and of 0.2 and 3.8 °C (Vancouver) by the 2100 s.

The extreme heatwaves’ durations strongly increase between the three time periods, especially the one of the longest heatwaves. The increase is more pronounced between 2100 s and 2050 s than between 2050 s and 2010s. By the 2010s, the duration of both the most intense and longest extreme heat waves is generally around one to three weeks, depending on the city. However, by 2050 s, the extreme heatwaves last more than a month in Abu Dhabi (41 days), Rome (42 days), and Stockholm (37 days), between 6 and 24 days for the most intense heatwaves in the other cities, between 7 and 49 days for the longest heatwaves in the other cities. By the 2100 s, in the five hottest cities (from climate zones 0 A, 0B, 1 A, 2 A, and 3 A), the longest and the most intense heatwaves last 3 to 4 months. This high number is found because the temperatures will constantly be above the current thresholds during the hot period of the year. In other parts of the world, the longest heatwave will be between three weeks and 2 months long by the 2100 s, except in Buenos Aires. For climate zone 3 A, the severity and duration of the heat waves in Rome are more significant than in Buenos Aires. This disparity might be attributed to the heatwave data record, which shows European cities have more exposure to heatwaves^19^. As expected, we observe that the durations of extreme intense heatwaves are generally shorter than the longest heatwaves.

### Effect of future TMY and HWY weather files on building performance

**Fig. 8:**
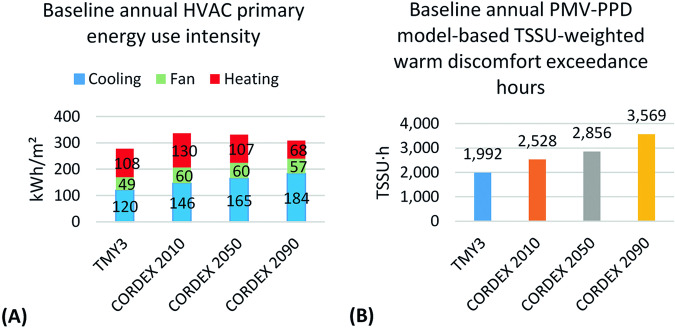
Effect of future TMYs on energy use (**A**) and summer thermal discomfort (**B**) in an air-conditioned single-family home in Los Angeles (Lee and Levinson^62^).

Lee and Levinson^62^ evaluated the effect of cool envelope strategies on heating, ventilation, and air conditioning (HVAC) primary energy use intensity and thermal comfort for a mechanically cooled single-family home in Los Angeles in Fig. 8. They used the future TMYs produced based on the methodology introduced in this paper (named CORDEX 2010, 2050, and 2090) as well as the historical Typical Meteorological Year 3 (TMY3), which spans 1991–2005^63^. Panel A shows that cooling demand grows over time. They also calculated the thermal sensation scale unit (TSSU) weighted warm discomfort exceedance hours (TSSU·h) to evaluate the Predicted Mean Vote (PMV) based thermal comfort, which is the sum of summer thermal discomfort when PMV exceeds + 0.7 according to ISO 1772-2:2018^64^. PMV greater than +0.7 is considered uncomfortably warm during the summer season according to ISO 17772-1:2017 Annex H.1 Category III^65^. Annex H.1 Category III, considered uncomfortably warm during the summer season. Panel B shows that the occupants experience many more TSSU-weighted warm-discomfort exceedance hours in the future because the cooling system is sized based on historical TMY3 weather, which results in many hours during which the cooling system cannot meet future loads. They also show that use of passive strategies such as cool envelope materials, helps decrease these loads. These results emphasize the need to use future TMYs to anticipate an increase in cooling energy use intensity and take necessary action to adapt building design or refurbishment to future climate.

**Fig. 9:**
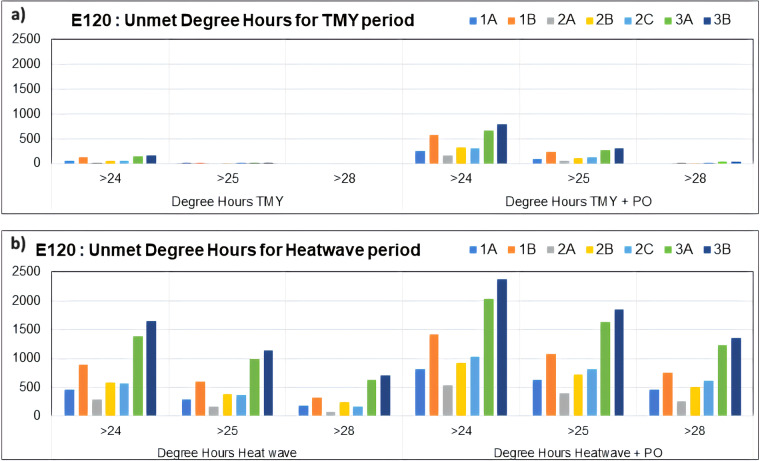
(**a**) Impact of future TMYs and (**b**) Impact of future HWYs on summer thermal discomfort from Sengupta *et al*.^66^.

Another example of how these weather files can be used is the work of Sengupta *et al*.^66^ in which they evaluated the overheating of an educational building in Ghent, Belgium, under future weather files, comparing the results with the future TMY and HWY prepared in this paper. Educational buildings in Belgium are not equipped with mechanical air conditioning, and recent heat waves have already posed a threat to occupants’ cognitive performance and health conditions. In their paper, they analyzed the thermal resilience of test lecture rooms with open windows at night for natural ventilation to flush heat and equipped with indirect evaporative cooling to cool the air during the daytime. Figure 9 shows the results of unmet degree hours (UDH) for different weather files: a) TMY and b) HWY (1 A: 2010s intense HW, 1B: 2010s severe and longest HW, 2 A: 2050 s intense HW, 2B: 2050 s severe HW, 2 C: 2050 s longest HW, 3 A: 2100 s intense HW, 3B: 2100 s severe and longest HW) with and without power outage (PO). The results emphasize that the HWYs present a much larger number of UDH when compared to TMY. The variety of HWY shows that HWY 1B leads to many UDHs due to its length of 28 days, while HWs of the 2100 s also predict a very elevated number of UDHs due to the increase in outdoor temperatures (Fig. 7). Additionally, a study by Sengupta *et al*.^67^ identifying, quantifying, and comparing different shocks that can increase overheating risk in buildings (e.g., outdoor shocks such as heatwaves and mechanical shocks such as solar shading failure, cooling strategy failure, natural night ventilation failure) proves that heatwaves are by far the most intense shocks for buildings that impact the thermal resilience to overheating. Thus, assessing and improving the buildings’ performance against heatwaves are a crucial step to future proof these buildings, emphasizing the robust methodology needed to develop and utilize future weather data and heatwave data to assess and design buildings.

## Usage Notes

The provided typical meteorological years (TMY) and years containing heatwaves (HWY) in both EPW format are ready-to-use weather datasets to perform building performance simulations using Energy Plus, TRNSYS, or any other building energy model. They permit assessment of the thermal performance of buildings under typical and extreme future climate scenarios. Therefore, they help evaluate the efficiency and resilience of building renovation solutions to climate change in different climate zones^68^. In particular, the TMY can be used to analyze changes in building heating and cooling loads under typical future weather conditions. The HWYs allow prediction of building thermal response under extreme heat events, which will be one major issue in the next decades. The multi-year (MY) datasets are also provided in CSV format to allow other authors to test different methods for assembling different types of future typical or extreme weather files for building performance assessments or in other sectors.

The provided datasets were generated based on the bias-corrected climate model MPI-ESM-LR/REMO, whose temperature projections are found to be the closest to the median of all climate model projections^69^. At least two other GCM/RCM model combinations satisfy the required spatial and temporal resolutions in the CORDEX database to generate weather files for building thermal performance analysis. These are the HadGEM2-ES/REMO and the NorESM1-M/REMO. Therefore, the results of this paper can be further expanded by comparing the outputs of all available CORDEX models. This can be used in future work to enrich the datasets. The datasets were generated based on RCP 8.5 climate projections, the worst-case socioeconomic scenario at the time of the IPCC AR5, and the most realistic based on the past and current emissions of greenhouse gases by the global community^70^. This means that they are suitable for applications in studies of system resilience, but they should be used with caution in building retrofits and HVAC system designs to avoid system oversizing or under-sizing.

It is possible to assemble additional weather files for other cities worldwide using other climate models with the same methodology as provided in this paper. The Python code to assemble the datasets from CORDEX climate projections is provided in the section “Code Availability.” Additional weather variables, such as cloud cover, precipitation, and longwave solar irradiance, would be an added value in the datasets. However, these additional variables were not currently available for all cities, neither observation. Indeed, robust climate observations are needed, and in this study, for some cities, only a few years (<10 years) of observations for the bias correction were available, which can affect the final result to an important extent for those cities. Future climate projections available on the CORDEX platform, with the newest SSP scenarios from CMIP6, might allow the include additional climate data in the datasets. In that case, observational data for these specific variables must also be found to correct the model.

Given that the multi-year datasets are provided, they could be used to select heatwaves based on methods different from the one chosen here. The common method used detects heatwaves solely based on the temperature; however, in some hot and humid parts of the world, humidity is known to be an important variable affecting indoor heat stress. The simple method proposed here was validated for several cities in France and allows a standardized approach that fits the purpose of a common method for all cities, climates, building typologies, and other local specificities. Nevertheless, the multiyear datasets allow the use of additional criteria to select the heatwaves with different methods. Beyond a different method, less future extreme heatwaves could also be selected for building design^4^.

As explained in the “boundary conditions” section, the datasets do not incorporate urban effects. In the selected GCM/RCM-REMO model, urban areas are represented as simple impervious surfaces. Recent studies have shown that a more detailed urban parametrization allows a better understanding of the regional-urban climate interactions and urban climate effects, such as UHI intensity^71–74^. However, this entails a significant increase in computing power and time, limiting the analysis to shorter time periods. Due to such limitations in modelling urban areas, the RCM REMO model does not accurately simulate climate modifications induced by urban features, such as the urban heat island effect or urban microclimates. Accordingly, bias-correction of the model projections was performed using observational data from weather stations located outside cities. The urban heat island effect and other urban climate modifications can be added to the weather datasets following different methodologies already proposed in building performance simulation studies^75–78^. Most climate models do not explicitly model urban areas and, at best, describe them as rock covers. Nonetheless, the very high resolutions reached now by the regional climate models may justify and require a more realistic parameterization of surface exchanges between urban canopy and atmosphere.

To quantify the potential impact of urbanization on the regional climate and evaluate the benefits of a detailed urban canopy model compared with a simpler approach, a sensitivity study was carried out over France at a 12 km horizontal resolution with the ALADIN-Climate regional model for 1980–2009 time period. Different descriptions of land use and urban modeling were compared, corresponding to an explicit modeling of cities with the urban canopy model TEB, a conventional and simpler approach representing urban areas as rocks, and a vegetated experiment for which cities are replaced by natural covers. A general evaluation of ALADIN-Climate was first done, which showed an overestimation of the incoming solar irradiance but satisfying results in terms of precipitation and near-surface temperatures. The sensitivity analysis then highlighted those urban areas had a significant impact on modeled near-surface temperature. A further analysis of a few large French cities indicated that over the 30 years of simulation, they all induced a warming effect both at daytime and nighttime with values up to +1.5 °C for the city of Paris. The urban model also led to regional warming extending beyond the boundaries of urban areas. Finally, the comparison to temperature observations available for the Paris area highlighted that the detailed urban canopy model improved the modeling of the urban heat island compared with a simpler approach.

The urban heat island effect could be added to the weather datasets by using offline urban canopy tools like the Urban Weather Generator (UWG)^79,80^, the Surface Urban Energy and Water Balance Scheme (SUEWS)^81^ module of the Urban Multi-scale Environmental Predictor (UMEP) GIS tool, or other similar urban canopy models^82^. Urban canopy models can also be coupled with mesoscale models such as the Weather Research and Forecasting (WRF) Model^83,84^ or the Global Environmental Multi-scale (GEM) Model^85^ for a better consideration of the urban boundary layer conditions^86^. The UWG is an easy-to-use, computational inexpensive tool that directly outputs urban weather files. However, it assumes that the city’s urban fabric is homogeneous and that the city is surrounded by rural areas. This can make its results inaccurate for coastal cities or inhomogeneous urban fabrics^87,88^. UWG accuracy may also be limited by the simplified ways in which it calculates latent heat balance flux and urban canyon wind speed^80^. Recently, new stand-alone UCM models have been developed that overcome some of the UWG limitations, such as the Stand-alone Urban Energy/Climate Model (SUECM)^89^. City Fast Fluid Dynamics (CityFFD)^90^ and the Vertical-city Weather Generator (VCWG)^91^. Machine learning techniques were also used to interpolate weather data spatially^92–94^. Any of these tools can be used to add urban effects as well as the evolution of land use to both the present and future TMYs and heatwave weather files presented in this data paper.

## Data Availability

The source codes to generate these datasets from CORDEX climate data can be found at: https://zenodo.org/record/7300024#.ZBbi4XbMI2x^95^.
