# Author Correction: Typical and extreme weather datasets for studying the resilience of buildings to climate change and heatwaves

**DOI:** 10.1038/s41597-025-04420-2

**Published:** 2025-01-15

**Authors:** Anaïs Machard, Agnese Salvati, Mamak P. Tootkaboni, Abhishek Gaur, Jiwei Zou, Liangzhu Leon Wang, Fuad Baba, Hua Ge, Facundo Bre, Emmanuel Bozonnet, Vincenzo Corrado, Xuan Luo, Ronnen Levinson, Sang Hoon Lee, Tianzhen Hong, Marcelo Salles Olinger, Rayner Maurício e Silva Machado, Emeli Lalesca Aparecida da Guarda, Rodolfo Kirch Veiga, Roberto Lamberts, Afshin Afshari, Delphine Ramon, Hoang Ngoc Dung Ngo, Abantika Sengupta, Hilde Breesch, Nicolas Heijmans, Jade Deltour, Xavier Kuborn, Sana Sayadi, Bin Qian, Chen Zhang, Ramin Rahif, Shady Attia, Philipp Stern, Peter Holzer

**Affiliations:** 1 Scientific and Technical Building Research Centre (CSTB), Energy and Environment Department, Grenoble, France; 2 Laboratory of Engineering Sciences for the Environment (LaSIE), La Rochelle University, La Rochelle, France; 3 Architecture, Energy & Environment (AiE), Polytechnic University of Catalonia, Barcelona, Spain; 4 Brunel University London, London, UK; 5 Department of Energy, Politecnico di Torino, Torino, Italy; 6 National Research Council Canada, Ottawa, Canada; 7 Centre for Zero Energy Building Studies, Department of Building, Civil and Environmental Engineering, Concordia University, Montreal, Canada; 8 Faculty of Engineering, British University in Dubai, Dubai, UAE; 9 Institute of Construction and Building Materials, Technical University of Darmstadt, Darmstadt, 64287 Germany; 10 Centro de Investigación de Métodos Computacionales (CIMEC), UNL/CONICET, Santa Fe, 3000 Argentina; 11 Lawrence Berkeley National Laboratory (LBNL), Berkeley, California USA; 12 Laboratory for Energy Efficiency in Buildings, Federal University of Santa Catarina, Florianopolis, Brazil; 13 Fraunhofer Institute for Building Physics IBP, Valley, Germany; 14 Department of Architecture, Faculty of Engineering Science, KU Leuven, Belgium; 15 Building Physics and Sustainable Design, Department of Civil Engineering, KU Leuven, Belgium; 16 Department of Environmental Architecture, Faculty of Architecture and Planning, Hanoi University of Civil Engineering, Ha Noi, Viet Nam; 17 Buildwise, Brussels, Belgium; 18 University of Gävle, 801 76 Gävle, Sweden; 19 Department of the Built Environment, Aalborg University, Aalborg, Denmark; 20 China Southwest Architecture Design and Research Institute Corp. Ltd., Chengdu, China; 21 Sustainable Building Design Lab, Department UEE, Faculty of Applied Sciences, Université de Liège, Liège, Belgium; 22 Institute of Building Research and Innovation (IBR&I), Wien, Austria

**Keywords:** Mechanical engineering, Civil engineering, Energy infrastructure, Mechanical engineering, Civil engineering, Energy infrastructure

Correction to: *Scientific Data* 10.1038/s41597-024-03319-8, published online 23 May 2024

In this article the author’s name Marcelo Salles Olinger was incorrectly given as Marcello Salles Olinger. The original article has been corrected.

